# Optimization of an O_2_-balanced bioartificial pancreas for type 1 diabetes using statistical design of experiment

**DOI:** 10.1038/s41598-022-07887-w

**Published:** 2022-03-18

**Authors:** Anne Mouré, Sawsen Bekir, Elodie Bacou, Quentin Pruvost, Karine Haurogné, Marie Allard, Laurence De Beaurepaire, Steffi Bosch, David Riochet, Olivier Gauthier, Gilles Blancho, Jean-Paul Soulillou, Denis Poncelet, Grégoire Mignot, Philippe Courcoux, Dominique Jegou, Jean-Marie Bach, Mathilde Mosser

**Affiliations:** 1 Oniris, INRAE, IECM, USC 1383, 44300 Nantes, France; 2 SSR Pédiatriques ESEAN-APF France Handicap, Nantes University Hospital, Nantes, France; 3 Oniris, Nantes Université, INSERM, RMeS, UMR 1229, F-44000 Nantes, France; 4 CRTI, UMR 1064, INSERM, Nantes Université, 44000 Nantes, France; 5 ITUN, CHU Nantes, 44000 Nantes, France; 6 GEPEA, UMR CNRS 6144 FR, Nantes Université, 44000 Nantes, France; 7 Oniris, INRAE, StatSC, USC 1381, 44000 Nantes, France

**Keywords:** Tissue engineering, Type 1 diabetes, Biomaterials - cells

## Abstract

A bioartificial pancreas (BAP) encapsulating high pancreatic islets concentration is a promising alternative for type 1 diabetes therapy. However, the main limitation of this approach is O_2_ supply, especially until graft neovascularization. Here, we described a methodology to design an optimal O_2_-balanced BAP using statistical design of experiment (DoE). A full factorial DoE was first performed to screen two O_2_-technologies on their ability to preserve pseudo-islet viability and function under hypoxia and normoxia. Then, response surface methodology was used to define the optimal O_2_-carrier and islet seeding concentrations to maximize the number of viable pseudo-islets in the BAP containing an O_2_-generator under hypoxia. Monitoring of viability, function and maturation of neonatal pig islets for 15 days in vitro demonstrated the efficiency of the optimal O_2_-balanced BAP. The findings should allow the design of a more realistic BAP for humans with high islets concentration by maintaining the O_2_ balance in the device.

## Introduction

The transplantation of a bioartificial pancreas (BAP), in which pancreatic islets are enclosed and protected in an immuno-isolating biomaterial, is a promising therapy for type 1 diabetes that does not entail strong immunosuppression in patients^1^. The BAP would also overcome the hurdle of human organ shortage by allowing the use of alternative islet sources. In particular, neonatal pig islets (NPIs) seem very promising because their isolation process is easily transposable to a clinical application in terms of good manufacturing practices and the stability of the phenotype of these primary cells^2^. However, one of the main limitations to the clinical success of BAP is the limited O_2_ supply to the encapsulated islet cells^3–5^. The O_2_ balance in the BAP results from complex interactions between several parameters including O_2_ consumption by the encapsulated cells, O_2_ diffusivity through the encapsulating material, geometrical features of the device, local O_2_ partial pressure (pO_2_) at the transplantation site, and vascularization of the graft surface^6^.

Pancreatic islets are large cell clusters that consume high amounts of O_2_^7^ and are very sensitive to hypoxia^8^. In the pancreas, a partial O_2_ pressure (pO_2_) of 40 mmHg (5%) has been reported in the endocrine tissue^9^. The in situ pO_2_ value should even be higher in pancreatic islets directly perfused with O_2_-rich arterial blood (80–100 mmHg) through a dense capillary network. During the isolation process, islets lose their vasculature, and the immuno-isolating membrane of the BAP prevents direct revascularization of the cell clusters. As a consequence, cell survival only relies on the passive diffusion of O_2_ through the BAP capsule and the cell clusters. After islets transplantation, the O_2_ tension in the environment surrounding the graft is assumed to be approximately 40 mmHg in extravascular sites^4,10–12^. Before graft surface neovascularization takes place within the first 2 weeks, O_2_ tensions may be below 5 mmHg (< 1%), even in naked islet grafts^9,13^. Pancreatic islet culture under low O_2_ tension results in impaired insulin secretion when pO_2_ of the media is < 50 mmHg^12,14^ and islet cell death when pO_2_ is < 5 mmHg^4,12^. Furthermore, dying cells release molecules and signals that could trigger an unwanted pro-inflammatory response, likely contributing to early graft failure^15–17^.

BAP capsules can be categorized into microcapsules and macrocapsules based on their size. Although spherical microcapsules seem the most suitable geometry from the standpoint of O_2_ diffusion, macrocapsules have a major advantage in terms of safety, as they can be fully retrieved and replaced. Theoretical modeling has been used to predict O_2_ distribution and hypoxic regions in BAPs according to the device’s geometry, islet size and density, surrounding O_2_ tension, and O_2_ diffusion across encapsulating materials^4,6,12,18^. Several studies aimed to optimize the features of BAPs to achieve therapeutic efficiency and avoid hypoxia-induced damages^4,12^. It is estimated that approximately 10,000 islet equivalent (IEQ)/kg of body weight are needed to restore normoglycemia in type 1 diabetes patients^5,19^, even considering a high pO_2_ of 40 mmHg outside the device. This requires large devices of 27 to 150 m for hollow fibers^4,12^ or 211 to 600 cm^2^ for planar devices depending on their width^4,5,12^. These dimensions are not amenable for human clinical transplantation. Pancreatic islets need thus to be encapsulated at a higher density in the BAP to obtain a device size that is suitable for clinical application^5^. However, increasing islet density in the device further decreases the O_2_ availability in the BAP. Thus, designing strategies to improve the O_2_ supply is necessary^4,15,17^.

Many strategies have been developed to address O_2_ limitations in the BAP. They include use of hypoxia-resistant cells^20^, increased vascularization induction around the device^11,21,22^, increased O_2_ diffusivity through the encapsulating biomaterial^23,24^, and an exogenous O_2_ source^24–27^. One of the most promising strategies uses a gas delivery system developed by Beta O_2_ Technologies (Rosh-Haayin, Israel). In this system, O_2_ enriched gas is injected into the Beta air device to deliver O_2_ to islets that are macro-encapsulated in alginate through a diffusion membrane. This BAP can maintain normoglycemia for 7 months in diabetic rats and should enable a therapeutically efficient device of 100 cm^2^ in type 1 diabetic patients. However, this strategy requires daily O_2_ injections^28^.

We previously described the potential of a combination of utilization of the extracellular hemoglobin HEMOXCell with an implantable O_2_ generator composed of silicone-encapsulated calcium peroxide (silicone-CaO_2_), to supply O_2_ to alginate macro-encapsulated NPIs up to 7 days in a hypoxic environment^24^. Further optimization to fine-tune the O_2_ balance between a high islet density and the O_2_ supply capacity of the oxygenation strategy remains crucial to design a functional BAP to treat type 1 diabetes. The aim of the present study was to optimize the configuration of the BAP incorporating the innovative strategy of oxygenation (ISO) by using a Design of Experiments (DoE) approach, including factorial design and response surface methodology (RSM). These statistical tools methods provide efficient ways to understand the complex relationships between input factors and output responses in a biological system, including the interactions between factors^29^. Using the DoE methodology, we (i) validated the efficiency of the oxygenation strategy on alginate encapsulated MIN6 beta cell pseudo-islets, (ii) designed an optimal O_2_-balanced BAP with high islet density carrying the oxygenation strategy, and (iii) assessed the optimal BAP design on NPI viability, function, maturation, and hypoxic stress in vitro.

## Results

### Screening of O_2_ supply strategies for BAP containing MIN6 pseudo-islets (MPIs)

**Figure 1:**
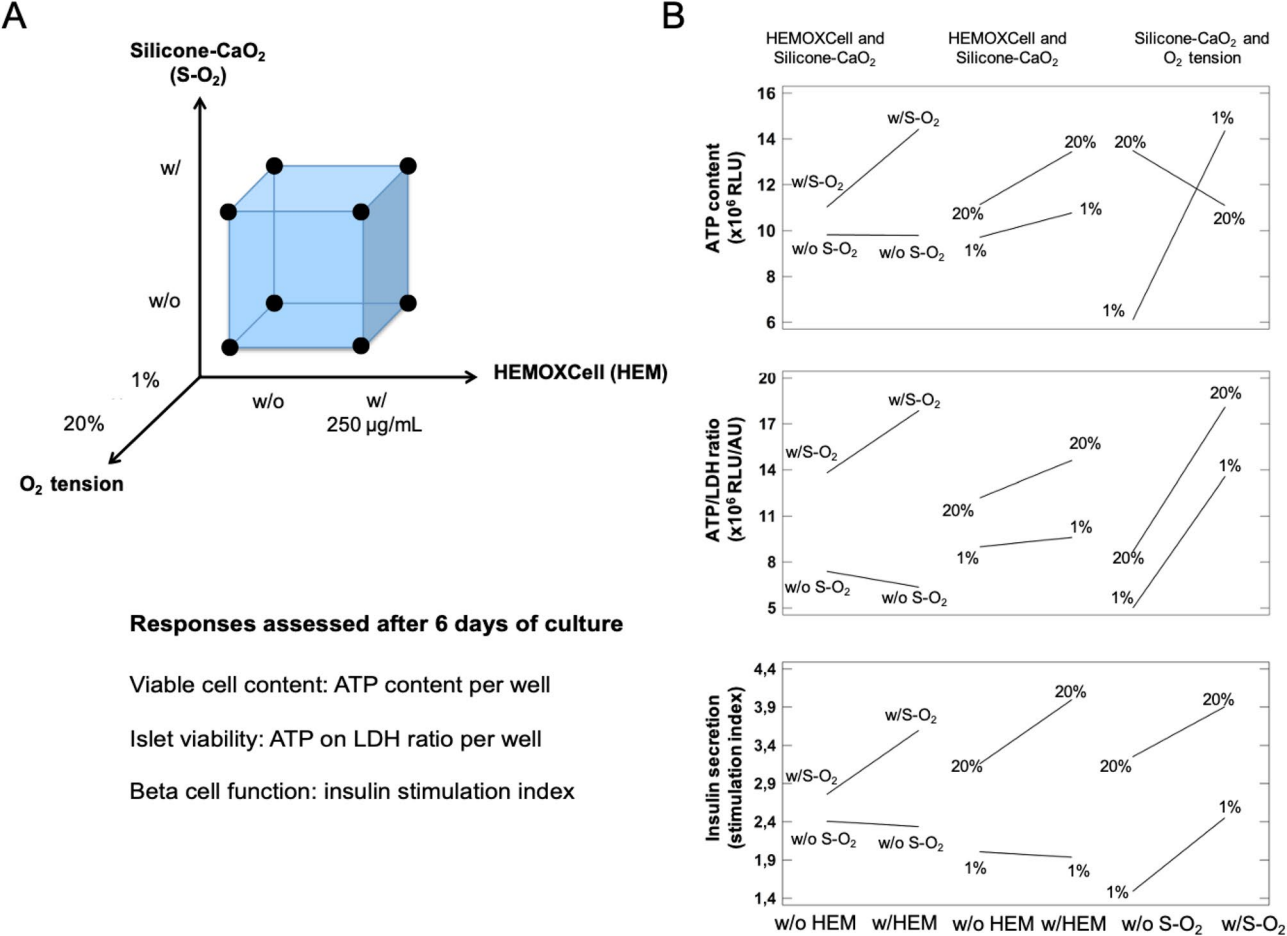
Screening experimental design. (**A**) The screening of the oxygenation strategies was done on MPIs encapsulated in alginate macrobeads using a full factorial design 2^3^. The three factors studied and their levels were: w/o or w/HEMOXCell (HEM), w/o or w/silicone-CaO_2_ (S-O_2_), and w/ 1% O_2_ or 20% O_2_ tension. The response variables assessed after 6 days of culture were the intracellular ATP content per well (RLU), ATP/LDH viability ratio per well (RLU/AU), and the insulin stimulation index. (**B**) Interaction plots of the screening DoE. Interactions involving HEMOXCell and silicon-CaO_2_, HEMOXCell and O_2_ tension, silicone-CaO_2_ and O_2_ tension are displayed concerning ATP content, ATP/LDH ratio, and insulin stimulation index.

The first objective of this study was to screen the selected O_2_ strategies (HEMOXCell and silicone-CaO_2_) in two different O_2_ tension environments (20% O_2_ and 1% O_2_) for their impact on the viability and function of encapsulated MPIs using a 2^3^ screening experimental design after 6 days of culture (Fig. 1A). The DoE was analyzed using variance analysis (Supplementary Tables 1–3). The models seemed to adjust well to the experimental data with determination coefficients (percentage of total variations explained by the model, R^2^) higher than 0.80 (Supplementary Tables 1–3). The experimental data obtained (Table 1) revealed the benefit of the presence of HEMOXCell on pseudo-islet viability, with a significant positive effect of this factor on the ATP content (p < 0.05, Table 2). The positive effects of the silicone-CaO_2_ disk on the ATP content and the ATP/LDH viability ratio were evident (p < 0.001, Table 2), while no significant improvement of the insulin stimulation index (p = 0.0633, Table 2). As expected, the hypoxic environment had a strong negative impact on pseudo-islet viability and function, with significant effects of O_2_ tension on ATP content, ATP/LDH ratio, and insulin index (p < 0.01, Table 2). A significant interaction was evident between silicone-CaO_2_ and the O_2_ tension on the ATP content (p < 0.001, Table 2). A strong positive effect of silicone-CaO_2_ on the MPI ATP content was observed in the hypoxic environment, while a negative effect of this factor was observed under normoxic conditions (Fig. 1B, silicone-CaO_2_ and O_2_ tension interaction). Interestingly, a significant positive interaction was evident between HEMOXCell and silicone-CaO_2_ on the ATP content (p < 0.05, Table 2), as was a benefit on the ATP/LDH viability ratio (p = 0.0890, Table 2). The effect of HEMOXCell was higher in combination with silicone-CaO_2_ than alone and vice-versa for these two responses (Fig. 1B). Even if not significant, we also observed a positive interaction between HEMOXCell and the O_2_ tension on the insulin stimulation index, with a higher effect of HEMOXCell in the normoxic environment. A multi-response optimization was performed to find the best condition to maximize pseudo-islet viability and function in the BAP in the hypoxic environment (Table 3). The best conditions under hypoxia were found in the presence of HEMOXCell and silicone-CaO_2_. These optimal conditions resulted in an increase of ATP content and ATP/LDH viability ratio by 14% and 48% respectively, while a decrease of insulin stimulation index by 19% was observed compared to the normoxic control without the ISO (Table 1).

**Table 1:** Factorial design matrix and experimental results obtained for the screening DoE.

Run	Factors			Responses^a^		
	HEMOXCell	Silicone-CaO_2_	O_2_ tension	ATP content (× 10^6^ RLU)	ATP/LDH ratio (× 10^6^ RLU/AU)	Insulin stimulation index
1	w/o	w/o	20% O_2_	13.52 ± 1.55	9.66 ± 4.42	3.1 ± 0.9
2	w/	w/o	20% O_2_	13.45 ± 2.64	7.78 ± 1.49	3.1 ± 0.1
3	w/o	w/	20% O_2_	9.09 ± 2.88	14.26 ± 1.45	3.1 ± 0.0
4	w/	w/	20% O_2_	13.43 ± 1.09	21.47 ± 6.16	4.5 ± 1.9
5	w/o	w/o	1% O_2_	6.11 ± 0.27	5.12 ± 1.72	1.7 ± 1.5
6	w/	w/o	1% O_2_	6.13 ± 0.59	4.94 ± 1.3	1.3 ± 0.8
7	w/o	w/	1% O_2_	13.98 ± 0.68	13.28 ± 8.81	2.2 ± 0.3
8	w/	w/	1% O_2_	15.41 ± 1.46	14.28 ± 4.99	2.5 ± 1.6

^a^Mean of three independent experiments.

**Table 2:** Estimated effects of factors and interactions and their statistical significance in the screening DoE.

Factors	ATP content (× 10^6^ RLU)		ATP/LDH ratio (× 10^6^ RLU/ AU)		Insulin stimulation index	
	Estimated effect ± SD	p-value*	Estimated effect ± SD	p-value*	Estimated effect ± SD	p-value*
HEMOXCell (HEM)	**1.68 ± 0.57**	**0.0109**	1.52 ± 1.39	0.2928	0.38 ± 0.36	0.3160
Silicone-CaO_2_ (S-O_2_)	**2.92 ± 0.57**	**0.0002**	**8.97 ± 1.39**	**< 10**^**–4**^	0.81 ± 0.39	0.0633
O_2_ tension	**2.05 ± 0.55**	**0.0027**	**4.11 ± 1.35**	**0.0095**	**1.61 ± 0.36**	**0.0013**
HEM and S-O_2_ interaction	**1.71 ± 0.57**	**0.0100**	2.55 ± 1.39	0.0890	0.45 ± 0.36	0.2404
HEM and O_2_ tension interaction	0.63 ± 0.55	0.2788	0.91 ± 1.35	0.5152	0.45 ± 0.36	0.2380
S-O_2_ and O_2_ tension interaction	**− 5.32 ± 0.55**	**< 10**^**–4**^	0.42 ± 1.35	0.7630	− 0.15 ± 0.36	0.6838

*p-values are computed from the analysis of variance performed for each response (Supplementary Tables 1–3). Statistical significance at 5% are highlighted in bold.

**Table 3:** Multi-response optimization for the screening DoE.

Optimization	Optimal level of factors			Response values at the optimum		
	HEMOXCell	Silicone-CaO_2_	O_2_ tension	ATP content (× 10^6^ RLU)	ATP/LDH ratio (× 10^6^ RLU/AU)	Insulin stimulation index
Optimization in hypoxia	w/	w/	Hypoxia	15.74	15.16	2.6

### Optimization of the BAP design regarding O_2_ balance

**Figure 2:**
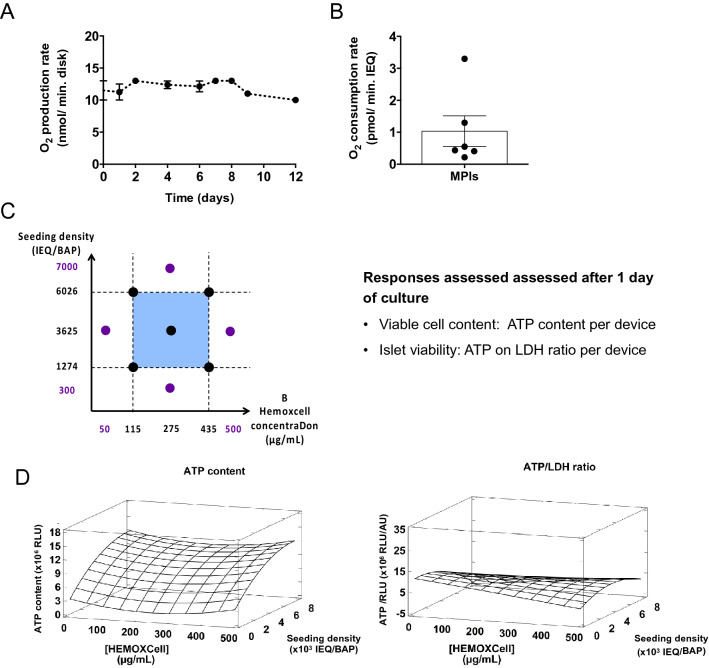
Optimization experimental design. Based on (**A**) the silicone-CaO_2_ O_2_ production rate and (**B**) the MIN6 pseudo-islet O_2_ consumption rate, (**C**) a central composite design was used to optimize the HEMOXCell concentration and islet seeding density in the BAP. The response variables, intracellular ATP content (RLU), and ATP/LDH viability ratio (RLU/AU) per device were assessed for MPIs encapsulated in alginate sheets after 1 day of culture. Results of O_2_ production rate (n = 2) and consumption rate (n = 6) are presented as mean ± SEM of independent experiments. (**D**) Response surface analysis for the optimization DoE. The plots represent the effects of the HEMOXCell concentration, islet seeding density, and their interaction on the ATP content and the ATP/LDH ratio in the BAP after 1 day of culture under 1% O_2_ tension.

In the second part of the study, the objective was to optimize the configuration of the BAP carrying the previously defined O_2_ strategy in a hypoxic environment to increase the density of viable islets in the device. To incorporate the O_2_ strategy, the BAP was made of two sheets of alginate encapsulating islets and HEMOXCell. The sheets were placed on either side of the silicone-CaO_2_ disk. A central composite experiment was designed to maximize the density of viable islets in the BAP (ATP content and ATP/LDH ratio) by tuning the HEMOXCell concentration and the islet seeding density (Fig. 2C). As primary islets used in the BAP in clinical settings would not proliferate, we optimized the device configuration on MPIs over a short (24 h) period, where differences in MIN6 cell proliferation were negligible (Supplementary Fig. 1). A HEMOXCell concentration ranging from 50 to 500 µg/mL was defined according to the literature^24,30,31^. The islet seeding density range was chosen on the basis of the O_2_ balance in the BAP. One silicone-CaO_2_ was able to produce a mean of 11.9 ± 0.3 nmol/min of O_2_ over 12 days (Fig. 2A). MPI OCR was estimated to be 1.04 ± 0.48 pmol/min.IEQ (Fig. 2B), giving a maximal islet density of 11,500 IEQ that could be supplied with O_2_ per silicone-CaO_2_ disk. In the literature, OCR was typically found to be approximately 1.64 ± 0.36 pmol/min.IEQ for human pancreatic islets^28,32–35^ and 2.2 ± 0.42 pmol/min.IEQ for neonate pig islets^33,36^, suggesting a maximal islet density of 7300 and 5400 per silicone-CaO_2_ disk, respectively. Based on these estimations, we decided to evaluate the islet seeding density range of 300 to 7000 IEQ in the BAP device (Table 4).

**Table 4:** Factorial design matrix and experimental results obtained for the optimization DoE.

Run	Factors		Responses^a^	
	Islet seeding density (IEQ/device)	HEMOXCell concentration (µg/mL)	ATP content (× 10^6^ RLU)	ATP/LDH ratio (× 10^6^ RLU/AU)
1	1274	115	5.69 ± 2.85	12.70 ± 11.7
2	1274	435	5.75 ± 2.87	8.35 ± 2.35
3	6026	115	10.05 ± 1.39	3.32 ± 2.37
4	6026	435	10.93 ± 1.66	4.82 ± 2.58
5	300	275	0.87 ± 0.23	5.41 ± 3.49
6	3625	50	8.60 ± 1.78	9.40 ± 4.94
7	3625	500	9.60 ± 3.12	8.38 ± 5.34
8	7000	275	11.16 ± 3.25	4.48 ± 3.09
9 (central point)	3625	275	8.11 ± 1.71	11.86 ± 1.27
10 (central point)	3625	275	7.93 ± 2.80	9.58 ± 6.28
11 (central point)	3625	275	8.12 ± 4.05	11.93 ± 8.04
12 (central point)	3625	275	6.42 ± 3.92	5.31 ± 5.8

^a^Mean of five independent experiments.

The optimization DoE was analyzed using variance analysis (Supplementary Tables 4 and 5). The resulting models adjusted well to the experimental data regarding ATP content according to the high R^2^ value obtained (0.76, n = 6). In contrast, a low R^2^ value was observed for the ATP/LDH ratio (0.39, n = 5), suggesting that factors other than those tested are involved in the response variations observed. Nevertheless, the lack-of-fit tests remained non-significant for both ATP content and ATP/LDH ratio (Supplementary Tables 4 and 5). The analysis of diagnostic plots confirmed the aforementioned results concerning model validation with overall good prediction for the ATP content, while poorer results were obtained for the ATP/LDH ratio (Supplementary Fig. 2). This was consistent with the higher experimental variability for the ATP/LDH ratio of the raw data obtained (Supplementary Fig. 2).

The application of RSM resulted in the following regression equation, modeling the relationship between the response variables and the factors as a second-order polynomial function:

$${\text{Y }} = \beta_{0} + \beta_{{\text{A}}} {\text{X}}_{{\text{A}}} + \beta_{{\text{B}}} {\text{X}}_{{\text{B}}} + \beta_{{{\text{AB}}}} {\text{X}}_{{\text{A}}} {\text{X}}_{{\text{B}}} + \beta_{{{\text{AA}}}} {\text{X}}_{{\text{A}}}^{{2}} + \beta_{{{\text{BB}}}} {\text{X}}_{{\text{B}}}^{{2}} + \varepsilon ,$$

where Y is the response; X_A_ and X_B_ are the linear variables associated with factors A (HEMOXCell) and B (islet seeding density), X_A_^2^ and X_B_^2^ the quadratic variables associated with factors A and B, β the coefficient associated with these variables, and ε is the residual variation. Estimates of regression coefficients and response surfaces are presented for each biological parameter tested in Supplementary Table 6 and Fig. 2D. We observed a significant quadratic effect of HEMOXCell concentration on ATP content (p < 0.05, Table 5). As expected, a strong positive effect of the islet seeding density on ATP content was observed (p < 0.0001, Table 5). Moreover, a negative quadratic effect of the islet seeding density was also observed for this parameter (p < 0.01, Table 5). No significant interaction was observed between the HEMOXCell concentration and the islet seeding density in the device.

**Table 5:** Estimated effects of factors and interactions and their statistical significance in the optimization DoE (with silicone-CaO_2_).

Factors	ATP content (× 10^6^ RLU)		ATP/LDH ratio (× 10^6^ RLU/AU)	
	Estimated effect ± SD	p-value*	Estimated effect ± SD	p-value*
A HEMOXCell concentration	4.9 ± 7.63	0.5238	− 1.37 ± 2.49	0.5859
B Islet seeding density	**8.34 ± 7.64**	**0.0000**	5.41 ± 2.72	0.0537
AA	2.89 ± 1.30	0.0307	− 2.14 ± 4.27	0.6193
BB	− **3.49 ± 1.3**	**0.0098**	− 7.65 ± 4.39	0.0893
AB interaction	1.64 ± 1.51	0.9142	6.22 ± 5.12	0.2310

*p-values are computed from the analysis of variance performed for each response (Supplementary Tables 4 and 5) and statistical significance at 5% are highlighted in bold.

According to these data, increasing the islet seeding density increased the viable cell content in the BAP but reached a plateau for the higher densities tested (Fig. 2D). Moreover, high islet density also resulted in a decreased islet viability ratio in the device (Fig. 2D). Multi-response optimization was used to determine the optimal HEMOXCell concentration and islet seeding density to maximize the ATP content and the ATP/LDH ratio in the BAP (Table 6). To this end, a desirability function was defined to find the best compromise between the two response variables. The optimal BAP configuration was defined as 50 µg/mL of HEMOXCell and 3284 IEQ/device in the presence of the silicone-CaO_2_ disk in a hypoxic environment. According to the standard curves of ATP content from viable encapsulated MPIs, we estimated the corresponding number of viable islets in the optimized BAP (Supplementary Fig. 3). An estimated cell number equivalent to 3572 IEQ was viable after 24 h in the hypoxic environment with the O_2_ strategy. As a comparison, the multi-response optimization was also performed (Table 6) with data obtained from the same DoE performed in hypoxia without the silicone-CaO_2_ disk (Supplementary Tables 6, 7, 8 and 9). The analysis of the central composite experimental design without silicone-CaO_2_ was carried out in the same way as with the O_2_ generator (data not shown). In the absence of the O_2_ generator, the optimal BAP configuration was defined with the 500 µg/mL HEMOXCell and 375 IEQ/device (Table 6).

**Table 6:** Multi-response BAP optimization under hypoxia with and without silicone-CaO_2_.

Optimization	Optimal level of factors		Response values at the optimum	
	Factor A HEMOXCell conc	Factor B Islet density	ATP content per device (RLU)	ATP/LDH ratio (RLU/AU)
w/silicone-CaO_2_	50 µg/mL	3284 IEQ/device	8.73 × 10^6^ ≈ 3572 viable IEQ^a^	9.64 × 10^6^
w/o silicone-CaO_2_	500 µg/mL	375 IEQ/device	2.24 × 10^6^ ≈ 656 viable IEQ^a^	3.83 × 10^6^

^a^Number of equivalent viable and dead MPIs interpolated from the ATP content standard curve (Supplementary Fig. 3).

### Validation of O_2_ balanced BAP design on MPIs

**Figure 3:**
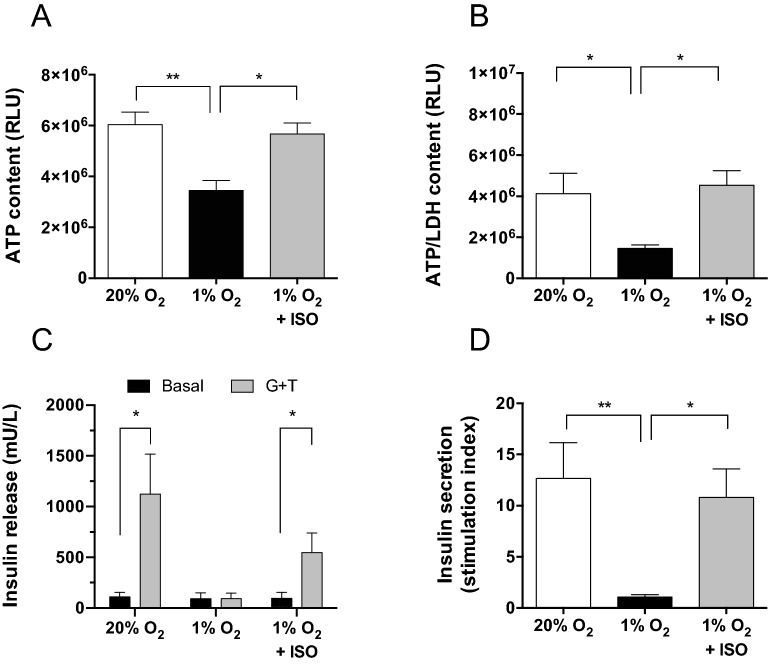
Viability and function of MIN6 pseudo-islets (MPIs) embarked in O_2_-balanced BAP. Alginate encapsulated MPIs (3000 IEQ/150 µL) were cultured for 3 days under 20% O_2_ (positive control, white) or 1% O_2_ condition without O_2_ strategy (negative control, black) or with innovative strategy of oxygenation (ISO) composed of silicone-CaO_2_ disk and 500 µg/mL HEMOXCell by alginate (1% O_2_ + ISO, grey). (**A**) Total metabolic activity (ATP content, RLU), (**B**) viability (ATP/LDH ratio, RLU/AU), (**C**) Glucose plus theophylline (G + T) responsive insulin release by 30 min sequential incubations of alginate encapsulated MPIs in basal medium (black bar) and glucose plus theophylline stimulation (grey bar), (**D**) Stimulation index (ratio of high glucose plus theophylline stimulation over basal insulin secretion). Results from independent experiments (n = 5–9) are expressed as mean ± SEM. *p < 0.05, **p < 0.01 (unpaired parametric t-test).

The optimal BAP configuration was first validated in vitro on MPIs in the hypoxic environment (1% O_2_) for 3 days. The seeding density of MPIs embarked in the O_2_-BAP was set to rounded value of the maximal density defined in the RSM optimization in presence of silicone-CaO_2_ (3000 IEQ) and the HEMOXCell concentration of 500 µg/mL was chosen. Indeed, as the oxygen released by the silicone-CaO_2_ will progressively decrease until exhaustion, the concentration of HEMOXCell determined in the RSM optimization plan performed without silicone-CaO_2_ was fixed. The results were compared to macro-encapsulated MPIs cultured without ISO in hypoxia (1% O_2_, negative control) or under high O_2_ tension (20% O_2_, positive control). As expected, adverse effects of hypoxia (1% O_2_) were observed on the MPI density and viability in the BAP without ISO as the ATP content and the ATP/LDH ratio decreased respectively by 43% (p < 0.01, Fig. 3A) and by 65% (p < 0.05, Fig. 3B) compared to the high O_2_ tension condition. In the case of the O_2_ balanced BAP (1% O_2_ + ISO), the ISO significantly increased the ATP content (p < 0.05) and the ATP/LDH ratio (p < 0.05) of MPIs compared to those cultured without. This allowed the ATP content and ATP/LDH ratio to reach the level observed in the positive control. Insulin secretion by the alginate encapsulated MPIs was assessed following glucose plus theophylline (G + T) stimulation after 3 days of culture (Fig. 3C,D). In high O_2_ tension, the production of insulin by MPIs after G + T stimulation was significantly increased (p < 0.01, Fig. 3C) and reached a stimulation index of 12.65 ± 3.52 (Fig. 3D). In hypoxia, the MPIs totally lost their ability to secrete insulin in response to G + T (1% O_2_). Interestingly, in the O_2_ balanced BAP, the adverse effect of hypoxia on the insulin-secretory function of MPIs was significantly prevented (p < 0.05, Fig. 3C,D). In fine, the O_2_-balanced BAP allowed reaching a stimulation index of 10.80 ± 2.78 close to the index observed in the positive control.

### Validation of O_2_ balanced BAP design on NPIs

Finally, the optimal BAP configuration was validated in vitro on primary NPIs in a hypoxic environment mimicking the acute hypoxic period (1% O_2_) before graft neovascularization for 15 days. As the maximal seeding density obtained using RSM methodology with MPIs was lower than the maximal theorical seeding density of NPIs that was estimated to be 5400 IEQ, we set as well the density of NPIs in the BAP to 3000 IEQ and the HEMOXCell concentration to 500 µg/mL. The results were compared to macro-encapsulated NPIs cultured without the O_2_ strategy in hypoxia (1% O_2_, negative control) or under high O_2_ tension (20% O_2_, positive control).

**Figure 4:**
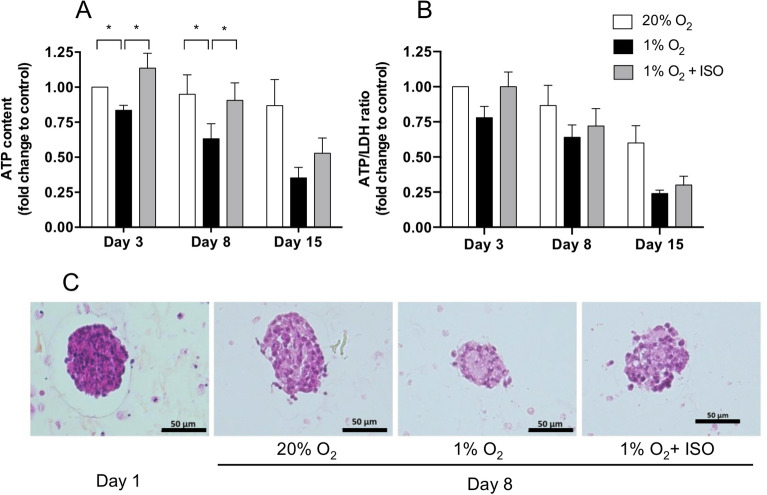
Viability of neonate pig islets embarked in O_2_-balanced BAP. Alginate encapsulated NPIs (3000 IEQ/150 µL) were cultured for 3, 8 and 15 days under normoxic condition (20% O_2_, positive control, white bar) or hypoxic condition without O_2_ strategy (1% O_2_, negative control, black bar) or with the innovative strategy of oxygenation (ISO) composed of silicone-CaO_2_ disk and 500 µg/mL HEMOXCell by alginate (1% O_2_ + ISO, grey bars). Fold change in (**A**) total metabolic activity (ATP content, RLU) and in (**B**) viability (ATP/LDH ratio, RLU/AU) of encapsulated NPIs compared to the control cultured at day 3 under 20% O_2_ without O_2_ strategy. Results from independent experiments (n = 4–6) are expressed as mean ± SEM (**A**,**B**). *p < 0.05 (Non-parametric Wilcoxon apparatus test). (**C**) Histological analyzes of formalin-fixed paraffin-embedded NPIs 4 µm thick cross-sections stained with hematoxylin–eosin-saffron on pre-encapsulated NPIs, 24 h after isolation (day 1) and on decapsulated NPIs after 8 days of culture within the BAPs**.**

As expected, adverse effects of hypoxia without the ISO on the NPI ATP content were observed compared to the high O_2_ tension condition on the different days of analysis (Fig. 4A). Indeed, while the total metabolic activity of NPIs under high O_2_ tension was almost completely maintained during the 15 days of culture, it decreased under hypoxia by 15% (p < 0.05), 37% (p < 0.05), and 63% (p = 0.0625) after 3, 8, and 15 days, respectively. Interestingly, in the case of the O_2_ balanced BAP, the ISO significantly increased the ATP content of NPIs on day 3 (p < 0.05) and 8 (p < 0.05) compared to NPIs cultured without the O_2_ strategy. This allowed the ATP content to reach that observed in the positive control. After 15 days, the O_2_ strategy still seemed to improve the NPI ATP content as a 25% increase was observed compared to NPI cultures in hypoxia without the O_2_ strategy (p = 0.1167). However, at this time, a drop of up to 40% ATP was observed compared to the NPIs cultured under high O_2_ tension. The viability (ATP/LDH ratio) of NPIs followed the same trends as observed for the total metabolic activity of NPIs under different conditions (Fig. 4B). Hypoxia decreased the viability of NPIs and the O_2_-strategy seemed to improve the viability. However, due to the high variability of LDH levels encountered in cultures, differences between groups were not found significant. The effect of O_2_ tension on NPI morphology was characterized using hematoxylin and eosin immunohistochemical staining after 8 days of culture (Fig. 4C). NPIs under hypoxia showed altered nuclei, while islets grown under hypoxia with the ISO had a similar morphology to those cultured under 20% O_2_.

**Figure 5:**
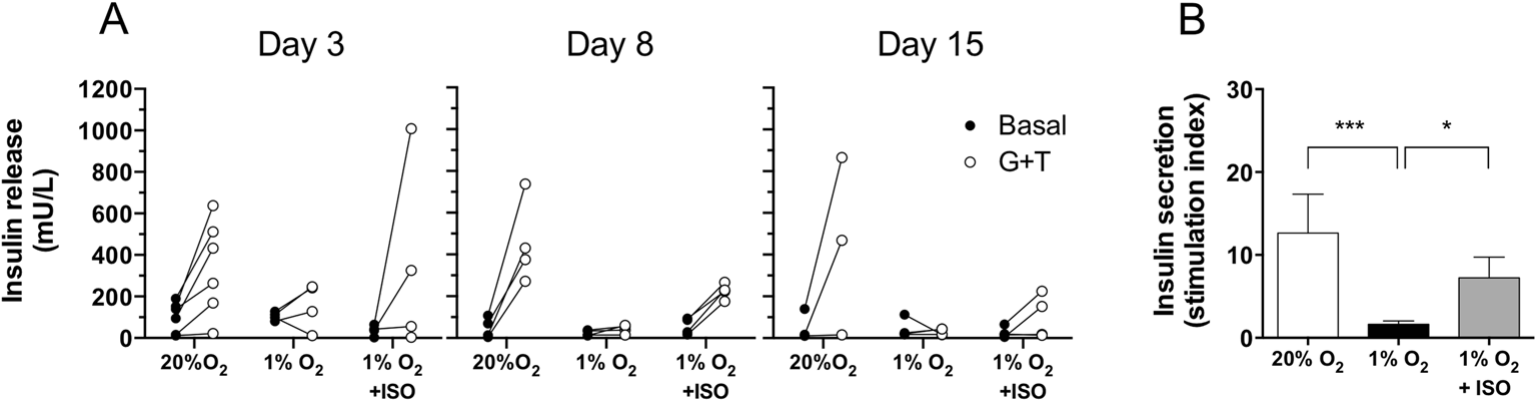
Function of neonate pig islets (NPIs) in O_2_ balanced BAPs. Alginate encapsulated NPIs (3000 IEQ/150 µL) were cultured for 3, 8, and 15 days under 20% O_2_ (positive control, white bar) or 1% O_2_ conditions without the O_2_ strategy (1% O_2_, negative control, black bar), or with the innovative strategy of oxygenation (ISO) composed of silicone-CaO_2_ disk and 500 µg/mL HEMOXCell by alginate (1% O_2_ + ISO, grey bars). (**A**) Glucose plus theophylline (G + T) responsive insulin release by 30 min sequential incubations of alginate encapsulated NPIs in basal medium (close circle) and glucose plus theophylline stimulation (open circle) after 3, 8 and 15 days of culture. (**B**) Stimulation indexes (ratio of high glucose plus theophylline stimulation over basal insulin secretion) of alginate encapsulated NPIs after 3, 8, and 15 days of culture. Results from independent experiments (n = 4–6) are expressed (**A**) individual results matched in basal or stimulation media or as (**B**) mean ± SEM. * p < 0.05, ***p < 0.005 (Mann–Whitney test).

Insulin secretion by the alginate encapsulated NPIs was assessed following glucose plus theophylline (G + T) stimulation after 3, 8, and 15 days of culture (Fig. 5). From day 3 of culture in hypoxia, the NPIs lost their ability to secrete insulin in response to G + T. In high O_2_ tension, NPIs remained functional until day 15. In the O_2_ balanced BAP, the adverse effect of hypoxia on the insulin-secretory function of NPIs seemed to be highly mitigated, although it failed in some experiments for which lower insulin responses were observed, even in NPIs cultured under high O_2_ tension (Fig. 5A). At any day of culture, a significant adverse effect was evident for NPI insulin stimulation indexes triggered by hypoxia from 13.5 ± 5.0 to 1.6 ± $0.4$ (p < 0.005). This was significantly reversed by the addition of the ISO to 7.2 ± 2.5 (p < 0.05) (Fig. 5B). The optimized O_2_ strategy attained a NPI stimulation index that was not significantly different (p = 0.262) from the NPIs cultured under high O_2_ tension.

**Figure 6:**
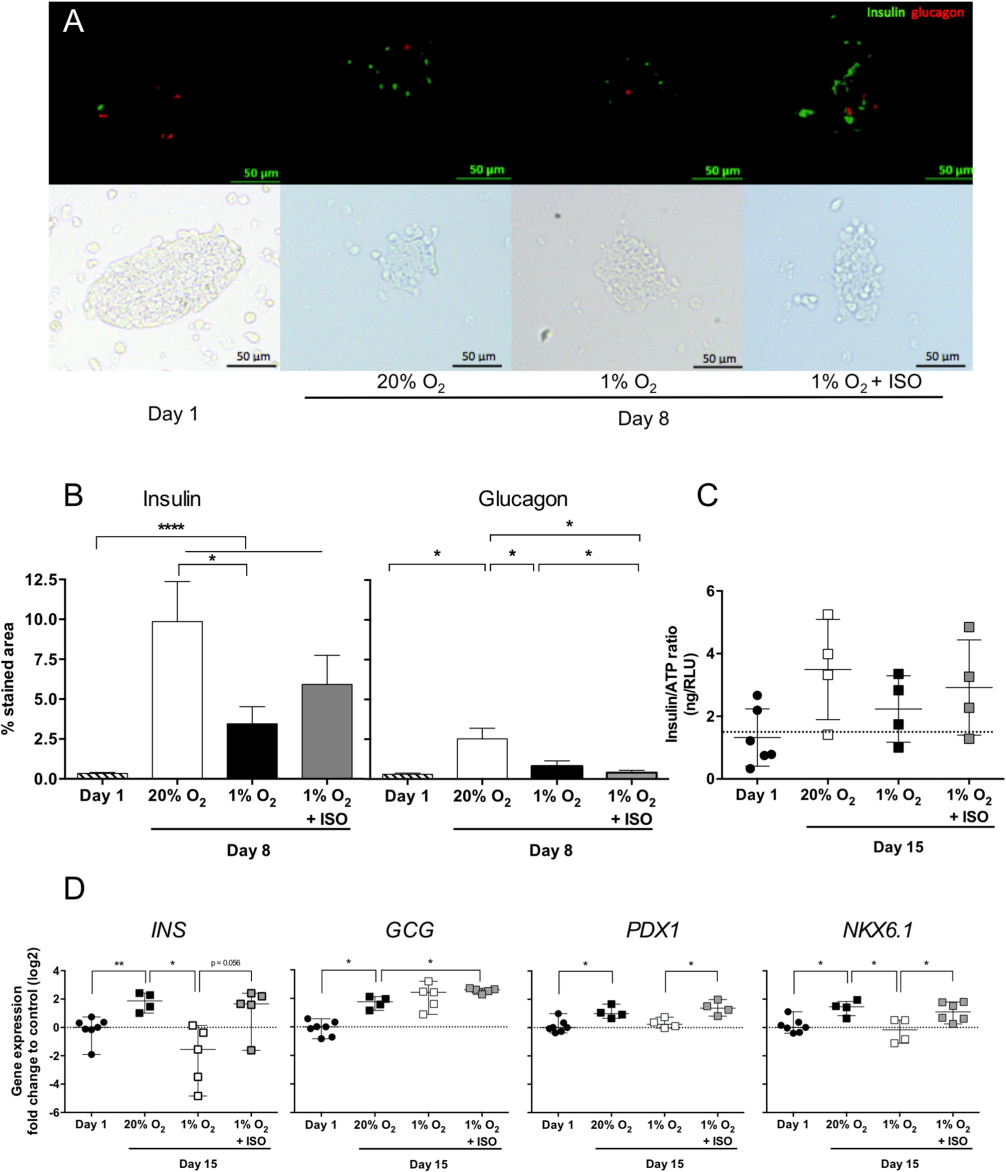
Effect of O_2_ strategy on the maturation of neonate pig islets (NPIs) embarked in O_2_ balanced BAPs. The analyses were performed on pre-encapsulated NPIs 24 h after isolation (day 1) and on encapsulated NPIs cultured for 8 or 15 days within BAPs in 20% O_2_ (positive control) and 1% O_2_ conditions without the O_2_ strategy (1% O_2_, negative control) or with the O_2_ strategy composed of silicone-CaO_2_ disk and 500 µg/mL HEMOXCell by alginate (1% O_2_ + ISO) (n = 4–7 pigs). (**A**) Immunostaining of insulin ß cells (green, Alexa-Fluor 488) and glucagon α cells (red, Alexa-Fluor 555) staining of NPIs in 4 µm thick cross-sections. Views in white light are shown to the lower-left of each photo. The scale is indicated in each picture. (**B**) Percentage of mean insulin-positive area per islet (Insulin) and percentage of mean glucagon-positive area per islet (Glucagon). The percentages of insulin and glucagon were quantified within 150 islets in day 1 (n = 4 pigs) and an average of 30 islets per condition after day 8 of culture (n = 3 pigs). (**C**) Intracellular insulin by ATP content ratio. (**D**) Relative quantitative RT-PCR expression analysis of insulin (*INS*), glucagon (*GCG*), pancreatic progenitor transcription factor (*PDX1*) and NKX6.1 (*NKX6.1*). *p < 0.05, ****p < 0.0005 (Unpaired parametric t-test or Mann–Whitney test).

The effect of O_2_ tension on NPI maturation was assessed by the quantification of the expression of insulin, glucagon, and PDX-1 after 8 or 15 days of culture compared to day 1 (Fig. 6 and Supplementary Fig. 4). The percentage of insulin-stained area in NPIs significantly increased from day 1 to day 8 regardless of O_2_ tension (p < 0.0001), although the observed increase was lower in 1% O_2_ compared to 20% O_2_ (p < 0.05, Fig. 6A,B). In addition, under 20% O_2_ conditions, we observed a maturation of alpha cells from day 1 to day 8 of culture, as shown by the enlarged glucagon-stained areas (p < 0.0001) and the low O_2_ pressure still seemed to hinder this phenomenon (p < 0.05, Fig. 6B). For both conditions, similar trends were observed in intracellular insulin/ATP ratio of NPIs (Fig. 6C) and in the percentage of PDX1 positive cells (Supplementary Fig. 4). We also performed quantitative real-time PCR for insulin, glucagon, *PDX1* and *NKX6.1* on NPI cultured in BAP for 15 days (Fig. 6D). All transcripts showed enhanced expression between day 1 and 15 days of culture in 20% O_2_ condition, confirming maturation and functionality gain over culture (p < 0.05). Except for glucagon, there was a drop in all studied transcripts expression when NPI were cultured in 1% O_2_ (p < 0.05 for INS and NKX6.1 and p = 0.057 for PDX1). Interestingly, the ISO significantly mitigated the hypoxic effect observed on PDX1 and NKX6.1 expression at the transcriptional level in NPIs (p < 0.05, Fig. 6D) with a trend to improve insulin transcript expression (p = 0.068, Fig. 6D) and protein (Fig. 6B,C). Surprisingly, ISO also caused glucagon expression in 1% O_2_-cultured NPI to rise beyond the level observed in 20% O_2_ condition after 15 days of culture (p < 0.05, Fig. 6D), while a decrease of the protein expression was observed after 8 days in culture (Fig. 6B). Altogether, these results underlined the influence of O_2_ tension on NPIs maturation and suggested the benefit of the oxygenation strategy to increase NPIs maturation in BAP after transplantation.

**Figure 7:**
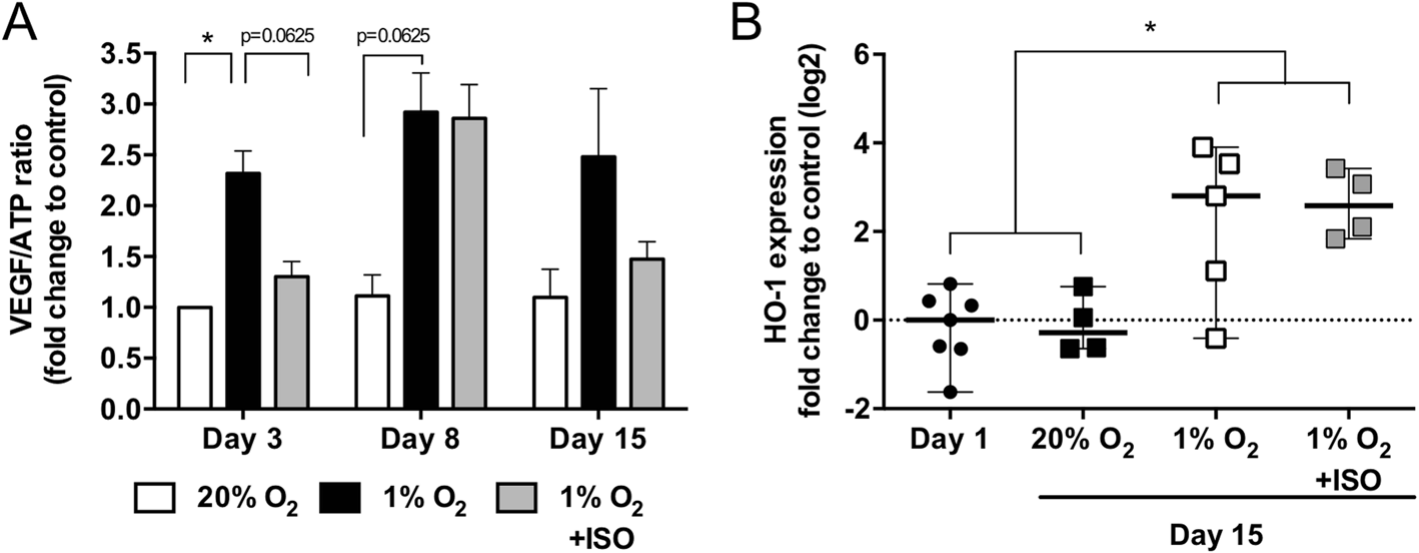
Hypoxic signature of neonate pig islets (NPIs) in O_2_ balanced BAPs. Alginate encapsulated NPIs (3000 IEQ/150 µL) were cultured for 3, 8, and 15 days under 20% O_2_ (20% O_2_, positive control, white bar) or 1% O_2_ condition without O_2_ strategy (1% O_2,_ negative control, black bar) or with the innovative strategy of oxygenation (ISO) composed of silicone-CaO_2_ disk and 500 µg/mL HEMOXCell by alginate (1% O_2_ + ISO, grey bars). Fold change in VEGF secretion by total metabolic activity (AU/RLU) of encapsulated NPIs compared to the control cultured for 3 days under 20% O_2_ without O_2_ strategy. (**B**) Relative quantitative RT-PCR expression analysis of Heme oxygenase (HO-1) of decapsulated NPIs cultured for 15 days within BAPs (n = 4–7). Results from independent experiments (n = 4–7) are expressed as mean ± SEM. *p < 0.05 (Non-parametric Wilcoxon apparatus test).

As part of the hypoxia response, we evaluated VEGF release by pancreatic islets in the O_2_-balanced BAP or negative and positive controls (Fig. 7A). As expected, hypoxia enhanced the VEGF/ATP ratio by 132% (p < 0.01) and 192% (p = 0.0625) after 3 and 8 days, respectively, compared to the positive control (NPIs cultured under high O_2_ tension) (Fig. 7A). At day 3, the hypoxia-driven VEGF production seemed to be prevented by ISO (p = 0.0625). With more time, however, no significant differences were observed between both conditions despite the oxygen supply. In addition, the relative expression of HO-1 in NPIs cultured for 15 days under the different conditions compared to day 1 was quantified to assess islet oxidative stress in response to hypoxia (Fig. 7B). The level of HO-1 mRNA expression in NPIs cultured under high O_2_ tension was stable from day 1 to day 15, while hypoxia increased HO-1 expression by 4 (p < 0.01). Despite the benefits previously observed on NPI viability, function, and maturation, the O_2_ strategy did not mitigate the effect of hypoxia on HO-1 expression, as similar mRNA levels were observed as in hypoxia.

## Discussion

Encapsulation in a biomaterial is essential to isolate transplanted cells from the immune or autoimmune response of the host and allows low or no immunosuppression regimens. However, encapsulation aggravates the O_2_ diffusional limitations. Low O_2_ tensions of approximately 10 mmHg are encountered in the graft after transplantation during the critical 7 to 14 day period preceding the BAP surface neovascularization^9,13^. This acute hypoxic period is responsible for massive pancreatic islet dysfunction and cell death^8,14^. A diffusion-based device design usually results in an inadequate transplant size with low encapsulated islet density, making it difficult to scale-up BAPs designed for small animals to large animal models or for human recipients^4^. Using the factorial design and RSM methodologies, we designed an O_2_-balanced BAP carrying islets at high density under low O_2_ tension (10 mmHg) by incorporating the combination of an O_2_ carrier and an in situ O_2_ generator.

MPIs were used to reduce the animal requirements for the optimization step of our O_2_-balanced BAP. The O_2_ strategy composed of HEMOXCell and the silicone-CaO_2_ disk was efficient to maintain macro-encapsulated MPIs in a viable and functional state. The presence of the O_2_ generator showed positive effects on both MPI viability and insulin secretion capacity in a hypoxic environment. On the contrary, the presence of the O_2_ generator under normoxic conditions resulted in a slight decrease in the viable cell content in the BAP. This might be due to the generation of hyperoxic stress within the islets cultured in normoxia with the O_2_ generator^37^ and/or to the production of toxic reactive oxygen species (ROS) produced by the silicone-CaO_2_ disk^24,26,38^. Interestingly, we also highlighted a significant positive effect of the presence of HEMOXCell on the viable cell content in the BAP. Of particular interest, the interaction between HEMOXCell and silicone-CaO_2_ showed a positive synergistic effect on the viability of MPI in the BAP. This could be explained by the anti-oxidant properties of the hemoglobin neutralizing the ROS produced by the silicone-CaO_2_ disk and by its capacity to increase the O_2_ diffusivity in the alginate hydrogel, thereby preventing hyperoxia^24^. Consequently, the best O_2_ strategy for the BAP carrying MPIs was the combination of the O_2_ carrier and generator.

Using this promising O_2_ strategy and the RSM methodology, we designed an O_2_ balanced BAP carrying a high density of viable islets for transplantation to sites with low O_2_ tension. Islet seeding densities exceeding 300 IEQ/cm^2^ in vitro decreases cell viability and function, and induces pro-inflammatory responses in human^17^ and rat^15^ islets. By increasing the O_2_ supply to the encapsulated islets with the combination of an O_2_ carrier and generator, we increased the pseudo-islet seeding density to 3284 IEQ/cm^2^ in the BAP composed of two 680 µm-thick alginate sheets (21,893 IEQ/mL) cultured in a hypoxic environment (1% O_2_). Maintenance of the O_2_ balance in the device allowed almost all the seeded MPIs to remain viable over the 24 h culture period. In contrast, the optimal seeding density without silicone-CaO_2_ was only 375 IEQ/cm^2^. Although the presence of HEMOXCell increased the viability of MPIs in the device, the tested concentrations of hemoglobin did not significantly affect the percentage of viable islets in the BAP. Thus, we selected the highest concentration of hemoglobin assessed to improve O_2_ diffusion throughout the BAP once the O_2_ generator becomes exhausted.

After the validation with MPI model, the ISO BAP configuration was extrapolated to BAPs containing primary pancreatic islets isolated from neonate pigs. NPIs remain the most promising alternative to human islets, as pig insulin and metabolic characteristics are very similar to human characteristics^39^. Moreover, naked NPIs exhibit natural resistance to hypoxia in terms of survival and function^20^. The endocrine portion of NPIs is immature and requires a long period before reaching functional maturity^22,40–43^. This maturation process may further be impacted by low O_2_ tensions in the graft^44,45^. Placing the presently designed BAP in an environment of low O_2_ tension to mimic the O_2_ tension in the graft before its vascularization resulted in a significant impairment of the NPIs viability and of their ability to secrete insulin in response to glucose plus theophylline. The discrepancy with previously published results might be explained by the negative effect of the macrocapsule and the high islet density on the O_2_ availability in the device versus the use of low-density naked NPIs. Hypoxia also prevented the NPI maturation process observed in the BAP cultured under high O_2_ tension, suggesting that the hypoxic environment before graft neovascularization should delay the time needed for NPIs to become functional in vivo. Interestingly, our BAP design including the oxygenation system mitigated the adverse effects triggered by hypoxia by improving NPI viability, function, and maturation. Nevertheless, the O_2_ strategy failed to prevent NPI upregulation of mRNA expression of the oxidative stress marker HO-1. This could be linked to the production of ROS by silicone-CaO_2_ disk^24,46^. Finally, VEGF was increased in response to hypoxia by NPIs. In the O_2_ balanced BAP, this pro-angiogenic response was maintained from days 3 to 8. We^24^ and other^30^ have already shown that hemoglobin may have a specific proangiogenic effect on pancreatic islets. The Hyperoxic–Hypoxic paradox^47^, suggesting that fluctuation in the free O_2_ concentration rather than the absolute level of O_2_ can be interpreted at the cellular level as a lack of O_2,_ could also explain the proangiogenic effect triggered by the oxygenation strategy. VEGF secretion could improve BAP engraftment by maintaining a beneficial proangiogenic signal in the absence of O_2_ limitation.

The DoE methodology was used to design an O_2_ balanced BAP allowing a high islet density by preventing the adverse effect of low O_2_ tension encountered in the graft before its vascularization. Use of such an O_2_ balanced BAP would lead to an acceptable device size of approximately 180 cm^2^ that would carry the 600,000 IEQ needed to achieve normoglycemia in adult human patient^5,19^. Moreover, the total number of islets required to reach therapeutic efficiency in humans might be decreased in this O_2_-balanced BAP^4,28,48,49^. In the past, the amount of islets necessary has been defined based on naked pancreatic human islets transplanted into the liver^19^. In this environment, islets are exposed to a hostile microenvironment leading to the rapid death of a large portion of the grafted cells^9,50–52^. By preventing immune reactions and hypoxia-induced damage by encapsulation and adequate O_2_ supply, the O_2_ balanced BAP could reduce post-transplant cell death and thus the number of pancreatic islets required per human patient. Finally, increasing the O_2_ tension in the BAP after transplantation promoted the differentiation and functional maturation of neonate pig islet beta cells in our settings. These results suggest that our oxygenation strategy may improve maturation defects of other promising alternative beta cell sources, such as insulin-producing cells derived from human stem cells^44,53^.

Our study has several limitations. Future work must focus on the in vivo evaluation of the efficacy of the O_2_ balanced BAP in diabetic mice in allo and xenotransplantation model. The use of genetically modified pig knockout for main xenoantigens, such as Neu5Gc and alpha1-3 GAL, could be useful to prevent the specific humoral response^54^ and to improve long term engraftment. Another important consideration is the capacity of the O_2_ regulated BAP to adapt to the varying O_2_ tensions encountered in the transplant site during the graft surface revascularization phase. Indeed, the O_2_ tension in the extravascular BAP is expected to rise from 10 mmHg (1% O_2_)^10,52^ after transplantation to 30 to 40 mmHg (5% O_2_) after revascularization of the device^10,11,55,56^. Presently, the O_2_ production rate from the silicone-CaO_2_ disk was almost constant over 12 days in vitro, suggesting that the O_2_ balance in the BAP should be maintained during the acute hypoxic period. Thereafter, the O_2_ supply from the vasculature may not be sufficient to maintain fully viable and functional cells in the BAP embarking islets at high density. The presence of HEMOXCell could improve the O_2_ supply from the graft vascularization during silicone-CaO_2_ depletion due to its capacity to potentiate the islet VEGF secretion, to increase O_2_ diffusivity through the alginate hydrogel^24^ and to better balance the O_2_ concentration in the device. The precise control and measurement of cell exposure to O_2_ are limited by O_2_ diffusion through the BAP and the high O_2_ uptake by islets. Further work is necessary to confirm the stability of HEMOXCell and its capacity to provide sufficient O_2_ from the vasculature to maintain the O_2_ balance in our high islet density BAP.

## Methods

The study was carried out in compliance with the ARRIVE guidelines.

### Animals and ethical concerns

The data were the collective results gathered from eight neonate pigs. Mini pigs aged 2 to 12 days were obtained from the INRAE PEGASE unit (Rennes, France). Experiments using pigs were approved by the Pays de la Loire Ethic Committee (Approval 01074.01/02) and were carried out in accordance with the relevant French (2013-118) and European regulations (2010/63 EU Directive). All efforts were made to minimize animal suffering and to restrict the number of experimental animals. Analgesia and anesthesia were provided by IM injection of methadone, midazolam and ketamine, and maintained with 2% isofluran. Piglets were subjected to laparotomy, and the pancreas was removed after exsanguination via the aorta causing euthanasia.

### Pancreatic islet isolation

NPIs were isolated from mini-pig pancreas as described previously^57^. Briefly, the pancreas was cut into 1 to 2 mm^3^ small pieces and digested with 25 mg/mL collagenase type V-S (Sigma-Aldrich, Saint-Louis, MO, USA) with gentle agitation for 14 to 16 min at 37 °C. The digest was filtered through a 500 µm pore size filter, washed in HBSS buffer (Biowest, ref L0612) supplemented with 0.5% (w/w) bovine serum albumin (BSA, Sigma-Aldrich), and then cultured in Ham’s F10 (Dutscher, Brumath, France) supplemented with 10 mM glucose, 50 mM IBMX, 2 mM l-glutamine, 10 mM nicotinamide, 100 IU/mL penicillin, and 100 mg/mL streptomycin. NPIs were cultured for at least 24 h in a normoxic condition (37 °C, 20% O_2_, 5% CO_2_) before encapsulation into alginate sheets.

### Pseudo-islet formation

The mouse MIN6 beta cell line was kindly provided by Pr. Jun-ichi Miyazaki (Osaka University Medical School, Japan)^58^. MIN6 cells were expanded in DMEM medium (Dutscher, Brumath, France) supplemented with 10% heat-inactivated calf serum (Invitrogen, Villebon-sur-Yvette, France), 1% penicillin/streptomycin/neomycin, and 50 µM 2-mercaptoethanol. To generate MIN6 pseudo-islets (MPIs), 10^6^ MIN6 cells/mL were cultured in non-treated culture petri dishes for 3 days at 37 °C in normoxic conditions.

### Alginate encapsulation

Clinical grade low viscosity and high guluronate sodium alginate 2.2% (w/v) (PRONOVA UP LVG, Novamatrix, Sandvika, UK), later called “hydrogel” was used for islet (NPIs and MPIs) encapsulation. Alginate was solubilized in 0.9% NaCl (w/v) by gentle stirring overnight at 4 °C and sterilized using 0.2 µm filtration. NPIs and MPIs were quantified using canonical standardized Islets Equivalent Quantities (IEQ)^59^. For encapsulation in macrobeads, islets were gently mixed in the hydrogel at 2500 IEQ/mL alginate. Macrobeads 3 mm in diameter were obtained by alginate extrusion through a 23 G needle using a syringe driver into a 100 mM CaCl_2_ gelation bath for 5 min. Alginate sheets (680 µm thickness, 1.2 cm diameter) containing varying islet concentrations (2000 to 46,667 IEQ/mL alginate) were prepared in 48-well plate (ref 150787, Thermofisher) lids by pouring 75 µL of the alginate suspension on the well surface on the lid. Crosslinking of flat alginate sheets was achieved by covering the lid wells with 0.22 µm filters (Merck Millipore, Burlington, MA, USA) associated with sintered glass filters (DWK Life Sciences, Wertheim, Germany) previously soaked in a 100 mM CaCl_2_ solution. A volume of 5 mL of CaCl_2_ solution was added to the top of the glass filter before a 7 min incubation at room temperature. After crosslinking, alginate beads or sheets were washed twice in 0.9% NaCl and then in NPI or MPI culture medium.

### Encapsulated islet culture

Eight macrobeads were placed in each P48-well plate well containing 500 µL of culture medium. Alginate sheets were cultured in 12-well plate inserts in 3 mL of culture medium (two sheets per insert) or in 24-well plates in 1.5 mL of medium (one sheet per well). Encapsulated islets were incubated either in a normoxic O_2_ tension environment (20% O_2_, 5% CO_2_, 37 °C) or in a hypoxia chamber (STEMCELL Technologies, Grenoble, France) filled with 1% O_2_ and 5% CO_2_ in N_2_ (37 °C) by purging the chamber at a rate of 20 L/min for 5 min as recommended by the supplier. Culture media and hypoxic atmosphere were renewed every 2 to 3 days of culture.

### Pseudo-islet viability assessment

Viable cell content in encapsulated islets was determined by intracellular ATP quantification (RLU, relative light unit) using the CellTiter-Glo^®^ 3D Cell Viability kit (Ref 69682, Promega, Charbonnieres-les-Bains, France) following the manufacturer’s recommendations. The ATP content standard curve for MPIs was obtained from pseudo-islets immediately after encapsulation in alginate patches at several densities. A linear correlation was observed between the luminescent signal of the CellTiter-Glo^®^ 3D Cell Assay and the fluorescent Cyquant DNA Assay (Ref C7026, Waltham, MA USA) regardless of the culture conditions. Cell death within the encapsulated islets was evaluated by quantifying the lactate dehydrogenase activity (Ref 11644793001, LDH, absorbance unit (AU), Roche, Meylan, France) in culture supernatants according to the manufacturer’s recommendations. ATP luminescence and LDH absorbance were evaluated on a FLUOstar OPTIMA luminometer (BMG Labtech, Champigny-sur-Marne, France).

### Insulin secretion assay

The capacity of encapsulated islets to secrete insulin following metabolic stimulation was evaluated by 30 min sequential incubations of alginate encapsulated islets in basal medium (RPMI [Ref 10043CV, PAA, Velizy-Villacoublay, Franc] containing 2 mM l-glutamine, 0.5% BSA, and 2.8 mM glucose), stimulation medium [basal medium supplemented with 20 mM glucose and 10 mM theophylline (Ref T1633, Sigma-Aldrish)], and basal medium. Secreted insulin concentrations were assessed in culture supernatants by ELISA (Ref 10124701 porcine insulin ELISA, Ref mouse insulin ELISA 10120001, Mercodia, Uppsala, Sweden,). Insulin secretion stimulation indexes were calculated as the ratio of the glucose + theophylline-stimulated insulin secretion level over the basal level of the encapsulated islets. Theophylline was used to potentiate insulin secretion as NPIs are immature islets containing insulin precursor cells, whose spontaneous secretion is notoriously low^57^.

### Intracellular insulin content

NPIs were recovered from the alginate sheet by incubation for 20 min at 37 °C in a decapsulating solution by calcium chelation in 5 mM citrate and 1 mM EDTA in PBS followed by mechanical dissociation and centrifugation. Proteins from naked or decapsulated NPIs were extracted by repeated pipetting and incubation steps at − 20 °C in 50 µL of an ethanol-HCl solution. Protein extracts were neutralized by adding 25 µL of Tris–HCl 1 M (pH = 7.5). Pig intracellular insulin was assayed in protein extracts by ELISA Absorbance was evaluated using a FLUOstar OPTIMA luminometer.

### Vascular endothelial growth factor (VEGF) quantification

VEGF secretion was assayed by ELISA in islet culture supernatants (Clinisciences, Nanterre, France).

### Transcriptomic analysis

Islets were recovered from alginate sheets as previously described and frozen at − 80 °C in NucleoZOL reagent (Macherey–Nagel, Düren, Germany). Total RNA was isolated according to the manufacturer’s instructions and reverse transcribed using MLV reverse transcriptase (Invitrogen, Carlsbad, CA, USA). Pig primer sequences as described earlier^24,60^ were purchased from Eurogentec (Angers, France). Validated TaqMan Gene Expression Assays (Thermofisher) were used for targets listed in Table 7. Real-time quantitative polymerase chain reaction (RT-qPCR) was performed on a CFX 96 Touch instrument (Bio-Rad, Hercules, CA, USA) using Hot FirePol qPCR reagents (Solis BioDyne, Tartu, Estonia). No template or samples processed without reverse transcriptase were included as negative controls. For each sample, the relative quantity was inferred from a standard curve created through the amplification of serial dilutions of a pool of representative samples. Whenever necessary, the target gene expression in the standard pool was artificially increased by spiking 5 µL of amplification product sequences diluted 1:1250. The expression of porcine genes encoding RPL19 (ribosomal protein L19) and PPIA (peptidylprolyl isomerase A) were used to normalize the expression of porcine PDX1 (pancreatic and duodenal homeobox 1), HO1 (heme oxygenase 1), NKX6-1 (NK6 homeobox I), INS (Insulin) and GCG (glucagon).

**Table 7:** Validated porcine primer and probe sets.

Gene name	Gene symbol	Thermofisher ID
Insulin	INS	Ss03386682_u1
Glucagon	GCG	Ss03384069_u1
NK6 homeobox I	NKX6-1	Ss03373352_m1

### Immunohistological analyses

#### Slide preparation

NPIs were recovered from alginate sheets as previously described, centrifuged, and fixed in paraformaldehyde (PFA; 4% (v/v)) for 30 min before being embedded in HistoGel™ (Thermo Fisher Scientific, Waltham, MA, USA). Morphology of formalin-fixed paraffin-embedded (FFPE) NPIs in cross-sections (3 µm) were analyzed using Hematoxylin–Eosin–Saffron stain.

#### Insulin and glucagon immunostaining

FFPE cross-sections of NPIs (3 µm) were incubated overnight with rabbit anti-insulin at 1:400 dilution (C27C9; Cell Signaling Technology, Beverly, MA, USA) and mouse anti-glucagon at 1:500 dilution (G2654; Sigma-Aldrich) IgGs, followed by 1 h with secondary Alexa-Fluor 488 donkey anti-rabbit IgG at 1:2000 dilution and Alexa-Fluor 555 donkey anti-mouse IgG at 1:1000 dilution as previously described^54^.

#### PDX-1 immunostaining

Slides were incubated overnight with horseradish peroxidase-conjugated anti-PDX-1 antibody at 1:500 dilution (219207-Abcam, Cambridge, UK). PDX-1 was visualized using EnVision + System-HRP, rabbit (DAB+) (K4011; Agilent, Santa Clara, CA, USA) and counterstained with hematoxylin. Neonate-pig-pancreatic sections were used as positive controls. Analyses without primary antibodies were performed as a negative control.

#### Insulin and glucagon stained area quantification

Images were acquired using an AxioVert microscope and Zen lite software (Carl Zeiss, Jena, Germany). Photos of representative fields of the slices were taken under both white light and fluorescence using the same exposure time for all images taken with the same staining. The percentage of insulin and glucagon staining per NPI area was quantified using ImageJ software (NIH, Bethesda, MD, USA).

### Pseudo-islet O_2_ consumption rate

The O_2_ consumption rate (OCR) by MPIs was measured in bioreactor experiments. OCR was determined on the first day of culture by placing 1000 IEQ MPIs in a P48 well (non-treated culture dish) in 500 µL of culture medium. Dissolved O_2_ concentration was assessed to the medium with a Clark electrode (InPro 6850i, Mettler-Toledo, Viroflay, France) inserted in the P48 plate wells and the Rhapsody software (Pierre Guerin Technologies, Niort, France). MPI OCR (pmol/min.IEQ) was calculated as the initial slope of the dissolved O_2_ concentration curve in the medium. A negative control without islets was performed in parallel.

### O_2_ supply strategies

The O_2_ carrier HEMOXCell hemoglobin (Hemarina, Morlaix, France) was mixed with the pseudo-islets in the alginate before crosslinking into macrobeads or sheets. The O_2_-generating biomaterial was prepared by mixing calcium peroxide (Sigma-Aldrich) in polydimethylsiloxane (silicone, Sylgard^®^ 184, Sigma-Aldrich) in a 1:3 ratio (weight/weight) as previously described by Pedraza^27^. A volume of 100 µL per well of silicone-CaO_2_ was degassed using vacuum bell and cross-linked in a P48 plate for 24 h at 60 °C.

### Silicone-CaO_2_ O_2_ production rate

The O_2_ production rate (OTR) of the silicone-CaO_2_ disks was followed during culture for 12 days by placing four silicone-CaO_2_ disks in 200 mL of PBS (Eurobio, Courtaboeuf, France) at 37 °C. The PBS was first deoxygenated by stirring at 200 rpm in a hypoxic atmosphere (N_2_). After reaching 0% O_2_, the container was sealed and the dissolved O_2_ concentration was measured in the PBS using a Clark electrode and Rhapsody software. OTR (nmol/min/disk) was calculated as the initial slope of the dissolved O_2_ concentration curve in the PBS. A negative control without a silicone-CaO_2_ disk was performed in parallel.

### Design of Experiment (DoE)

#### Screening

Screening of the oxygenation strategies was performed on MPIs encapsulated in alginate macrobeads using a full factorial design 2^3^ (Fig. 1). The influence of the main factors and their first-order interactions was analyzed. The three factors studied and their levels were: without/with HEMOXCell, without/with silicone-CaO_2_ and normoxic/hypoxic O_2_ tension. The response variables were intracellular ATP content per well (RLU), ATP/LDH viability ratio per well (RLU/AU), and insulin stimulation index. These response variables were assessed after 6 days of culture under the different conditions defined by the experimental design (Table 2).

#### Response surface method (RSM)

Based on the screening of the O_2_ strategies, RSM was used to optimize the BAP configuration concerning the O_2_ balance in the alginate sheet device. The objective was to maximize the density of viable MPIs in the BAP by tuning the HEMOXCell concentration and the islet seeding density in the hypoxic environment in the presence of the silicone-CaO_2_ disk. A central composite design (2^2^ factorial design with 4-star points and four replicates of the central point) was used to fit a second-order polynomial model (Fig. 2C). The model validation was performed by analyzing the lack-of-fit test results, the determination coefficient R^2^ value, and the diagnostic plots. The two factors studied were HEMOXCell concentration and islet seeding density, and their ranges were determined according to the literature and the determination of silicone-CaO_2_ OTR and pseudo-islet OCR. The response variables were intracellular ATP content (RLU) and ATP/LDH viability ratio (RLU/AU) per device. These response variables were assessed on pseudo-islets encapsulated in alginate sheets cultured for 24 h under the different conditions defined by the experimental plan (Table 5). The optimum values were determined by solving the regression equations and analyzing the response surface plots. A multi-response optimization was performed to achieve the best compromise to maximize viability and function of the encapsulated pseudo-islets.

### Statistical analyses

The experimental design for the screening and the optimization steps were repeated independently at least three times. Analysis of variance (ANOVA), regression analysis, and graphical display of DoE results were performed using the Statgraphics Centurion software 18.1.06. Assessment of the optimal BAP design on NPIs was performed on a minimum of four independent experiments. The significance of differences between groups was evaluated using a non-parametric test (Mann–Whitney or paired Wilcoxon tests) or a parametric unpaired t-test. A p-value < 0.05 was considered statistically significant. Graphs’ formatting was performed using Graphpad Prism software 8.0.2.

## Supplementary Information

Supplementary Information.

## Nomenclature

AU: Absorbance unit
ATP: Adenosine triphosphate
ANOVA: Analysis of variance
BAP: Bioartificial pancreas
BSA: Bovine serum albumin
DoE: Design of experiments
HO-1: Heme oxygenase 1
LDH: Lactate dehydrogenase
MPIs: MIN6 beta cell pseudo-islets
NPIs: Neonatal pig islets
OCR: O_2_ consumption rate
pO_2_: O_2_ partial pressure
OTR: O_2_ production rate
PDX1: Pancreatic progenitor transcription factor
PPIA: Peptidylprolyl isomerase A
RT-qPCR: Real-time quantitative polymerase chain reactions
RSM: Response surface methodology
IBMX: Isobutylmethylxanthine
IEQ: Islet equivalent quantities
RLU: Relative light units
ROS: Reactive oxygen species
RPL19: Ribosomal protein L19
VEGF: Vascular endothelial growth factor
