# Supplementary material for: Optimization of an O2-balanced bioartificial pancreas for type 1 diabetes using statistical design of experiment
Source: Sci Rep. 2022 Mar 18;12:4681. doi: 10.1038/s41598-022-07887-w (PMC8933496; doi:10.1038/s41598-022-07887-w)
Supplement: Supplementary file 1 — Supplementary Information. [file 41598_2022_7887_MOESM1_ESM.docx]

**Supplemental Figure**

**Supplemental Figure 1. MIN6 cell proliferation within encapsulated MPIs.** MIN6 cells were encapsulated at 500, 1500, and 3500 IEQ/cm^2^ in alginate sheets and cultured in the normoxic (20% O_2_) environment for 0, 1, 3, and 6 days (A to D). ATP content measurement allowed monitoring of MIN6 cell proliferation within the encapsulated MPIs over time. Linearity between fold increase in ATP content (mentioned in plots) and fold increase in islet density was maintained only on day 0 and 1 of culture, suggesting different proliferation rates according to islet seeding density during longer culture periods. Results are presented as the mean ± SEM.

**Supplemental Figure 2. Raw data from the optimization DoE** Raw data of ATP content (A) and ATP/LDH ratio (B) are represented according to the conditions tested in the optimization experimental plan without (black bars) and with (grey bars) the silicone-CaO_2_ disk of MIN6 pseudo-islets cultured under hypoxia (1% O_2_) for 24 h. Data are expressed as mean ± SEM of independent experiments (n=6-7).


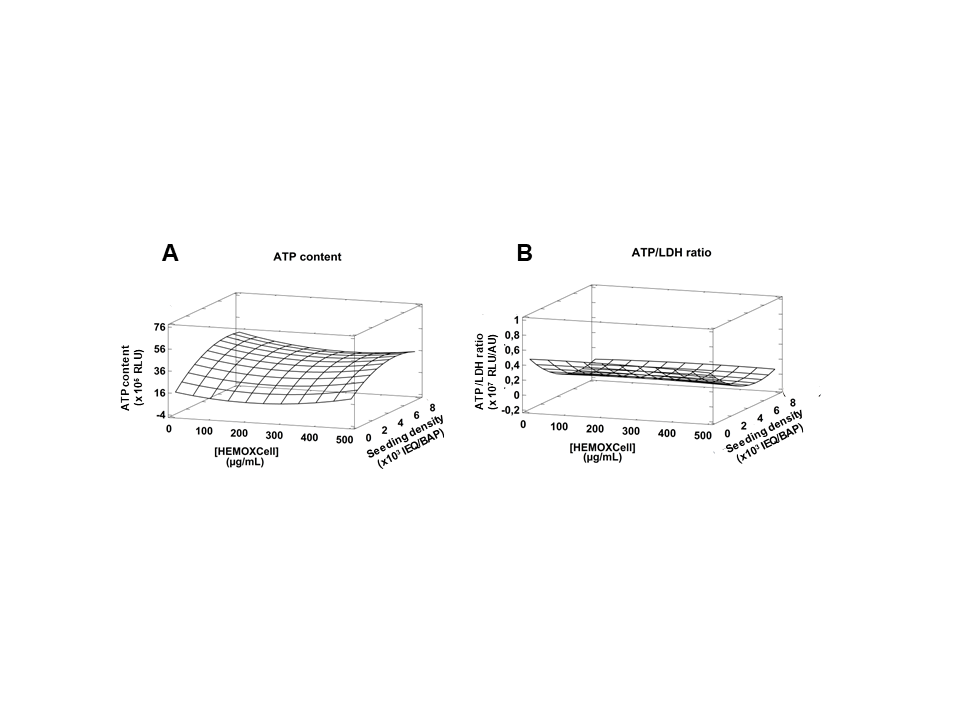


**Supplemental Figure 3. Response surface analysis for the control DoE (w/o silicon-CaO_2_)** The plots represent the effects of the HEMOXCell concentration, islet seeding density, and their interaction on the ATP content (A) and the ATP/LDH ratio (B) in the BAP without the Silicon-CaO_2_ disk after 1 day of culture under 1% O_2_. (n=7)

**Supplemental Figure 4. Standard curve of ATP content according to the MIN6 pseudo-islets seeding density in alginate sheets**. ATP content was measured on day 0? after encapsulation of MPIs in alginate sheets at densities ranging from 150 to 3,500 IEQ per sheet. Linear regression allowed the estimation of the quantity of viable islets in the BAP composed of two alginate sheets.

r = 0,57

r = 0,73

**20% O**_2_

**1% O**_2_

A

B

**Supplemental Figure 5. Standard curve of ATP content according to DNA quantity**. Correlation between ATP content and DNA quantity was measured on day 1 using NPIs under 20% O_2_  (A) and 1% O_2_ (B) (n = 13 by condition ; p<0,001 spearman)


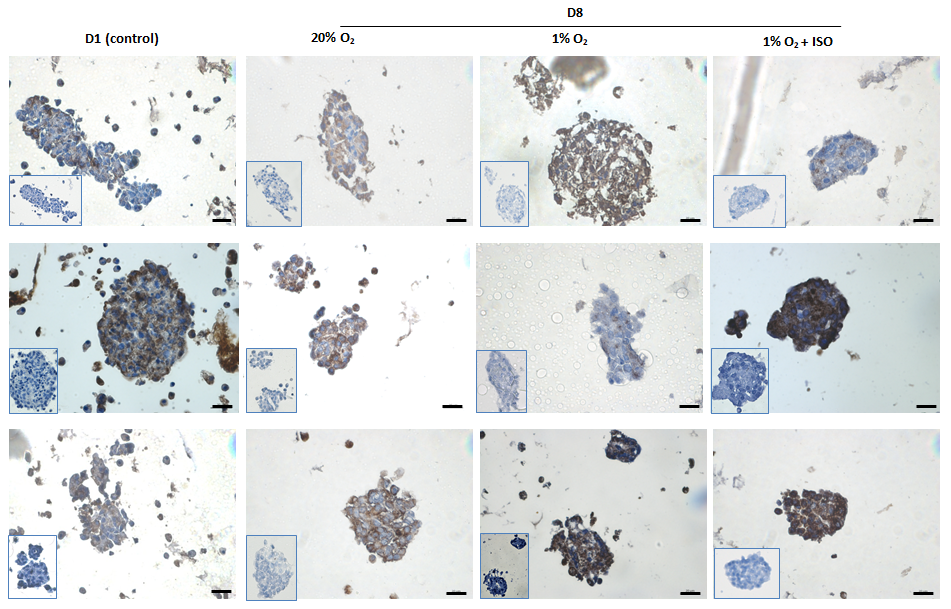


**Supplemental Figure 4. PDX-1 expression in neonate pig islets (NPIs) within BAPs.** Staining was performed on NPIs before encapsulation (day 1) and on NPIs recovered from the BAP through a de-encapsulation process after 8 days of culture under both positive and negative controls, respectively. Normoxia (20% O_2_) and hypoxia (1% O_2_) and under the hypoxic condition with the O_2_ strategy. The nuclei are counterstained blue with hematoxylin. In each column, the three captions correspond to three different neonate pigs. The lower left side picture on each caption corresponds to the negative control (no PDX-1 primary antibody). Scale bars: 20 μm.

**Supplemental Tables**

**Supplemental Table 1. Analysis of variance for ATP content from the screening DoE**.

| **Factors** | **Sum of squares**  **(× 10^11^)** | **Degrees of freedom** | **Mean square**  **(× 10^11^)** | **F ratio** | **p-value** |  |
| --- | --- | --- | --- | --- | --- | --- |
| HEMOXCell (HEM) | 145.81 | 1 | 145.81 | 8.80 | 0.0109 |  |
| Silicone-CaO_2_ (S-O_2_) | 439.51 | 1 | 439.51 | 26.53 | 0.0002 |  |
| O_2_ tension | 226.73 | 1 | 226.73 | 13.69 | 0.0027 |  |
| HEM and S-O_2_ interaction | 150.30 | 1 | 150.30 | 9.07 | 0.0100 |  |
| HEM and O_2_ tension interaction | 21.16 | 1 | 21.16 | 1.28 | 0.2788 |  |
| S-O_2_ and O_2_ tension interaction | 1526.42 | 1 | 1526.42 | 92.15 | < 10^-4^ |  |
| Block | 168.28 | 2 | 84.14 | 5.08 | 0.0234 |  |
| Residual error | 215.33 | 13 | 16.56 |  |  |  |
| **Total error** | 3134.30 | 21 |  |  |  |  |
| **Determination coefficient R^2^** | **0.93** | | | | | |

**Supplemental Table 2. Analysis of variance for ATP/LDH release from the screening DoE**.

| **Factors** | **Sum of squares**  **(× 10^11^)** | **Degrees of freedom** | **Mean square**  **(× 10^11^)** | **F ratio** | **p-value** |
| --- | --- | --- | --- | --- | --- |
| HEMOXCell | 118.75 | 1 | 118.75 | 1.20 | 0.2928 |
| Silicone-CaO_2_ | 4137.73 | 1 | 4137.73 | 41.88 | < 10^-4^ |
| O_2_ tension | 911.37 | 1 | 911.37 | 9.22 | 0.0095 |
| HEMOXCell and Silicon-CaO_2_ interaction | 333.71 | 1 | 333.71 | 3.38 | 0.0890 |
| HEMOXCell and O_2_ tension interaction | 44.22 | 1 | 44.22 | 0.45 | 0.5152 |
| Silicone-CaO_2_ and O_2_ tension interaction | 9.37 | 1 | 9.37 | 0.09 | 0.7630 |
| Block | 1504.12 | 2 | 752.06 | 7.61 | 0.0065 |
| Residual error | 1284.39 | 13 | 98.80 |  |  |
| **Total error** | 9010.97 | 21 |  |  |  |
| **Determination coefficient R^2^** | **0.86** | | | | |

**Supplemental Table 3. Analysis of variance for insulin stimulation index from the screening DoE**.

| **Factors** | **Sum of squares** | **Degrees of freedom** | **Mean square** | **F ratio** | **p-value** |
| --- | --- | --- | --- | --- | --- |
| HEMOXCell | 0.66 | 1 | 0.66 | 1.11 | 0.3160 |
| Silicone-CaO_2_ | 2.60 | 1 | 2.60 | 4.36 | 0.0633 |
| O_2_ tension | 11.65 | 1 | 11.65 | 19.56 | 0.0013 |
| HEMOXCell and Silicon-CaO_2_ interaction | 0.93 | 1 | 0.93 | 1.56 | 0.2404 |
| HEMOXCell and O_2_ tension interaction | 0.94 | 1 | 0.94 | 1.58 | 0.2380 |
| Silicone-CaO_2_ and O_2_ tension interaction | 0.11 | 1 | 0.11 | 0.18 | 0.6838 |
| Block | 7.08 | 2 | 3.54 | 5.94 | 0.0199 |
| Residual error | 5.96 | 13 | 0.60 |  |  |
| **Total error** | 30.53 | 21 |  |  |  |
| **Determination coefficient R^2^** | **0.81** | | | | |

**Supplemental Table 4. Analysis of variance for ATP content from the optimization DoE with the silicone-CaO_2_ disk**.

| **Factors** | **Sum of squares**  **(× 10^11^)** | **Degrees of freedom** | **Mean square**  **(× 10^11^)** | **F ratio** | **p-value** |  |
| --- | --- | --- | --- | --- | --- | --- |
| HEMOXCell concentration (HEM) | 37.95 | 1 | 37.95 | 0.87 | 0.3686 |  |
| **Islet seeding density (Islet)** | **3440.50** | **1** | **3440.50** | **79.11** | **< 10^-4^** |  |
| HEM & HEM | 144.06 | 1 | 144.06 | 3.31 | 0.0938 |  |
| Islet & Islet | 160.23 | 1 | 160.23 | 3.68 | 0.0790 |  |
| HEM & Islet | 13.73 | 1 | 13.73 | 0.32 | 0.5841 |  |
| **Block** | **1300.14** | **3** | **433.38** | **9.97** | **0.0014** |  |
| Lack-of-fit test | 708.58 | 27 | 26.24 | 0.60 | 0.8660 |  |
| Residual error | 521.88 | 12 | 43.49 |  |  |  |
| **Total error** | 6392.18 | 47 |  |  |  |  |
| **Determination coefficient R^2^** | **0.81** | | | | | |

**Supplemental Table 5. Analysis of variance for ATP/LDH ratio from the optimization DoE with the silicone-CaO_2_ disk**.

| **Factors** | **Sum of squares**  **(× 10^11^)** | **Degrees of freedom** | **Mean square**  **(× 10^11^)** | **F ratio** | **p-value** |
| --- | --- | --- | --- | --- | --- |
| HEMOXCell concentration (HEM) | 89.30 | 1 | 89.30 | 0.12 | 0.7367 |
| Islet seeding density (Islet) | 430.71 | 1 | 430.71 | 0.58 | 0.4658 |
| HEM & HEM | 16.24 | 1 | 16.24 | 0.02 | 0.8857 |
| HEM & Islet | 1269.91 | 1 | 1269.91 | 1.71 | 0.2234 |
| Islet & Islet | 311.01 | 1 | 311.01 | 0.02 | 0.5337 |
| Block | 3064.12 | 2 | 1532.06 | 2.06 | 0.1830 |
| Total error | 2842.79 | 19 | 149.62 | 0.20 | 0.9984 |
| Residual error | 6682.54 | 9 | 742.51 |  |  |
| Total error | 14827.60 | 35 |  |  |  |
| **Determination coefficient R^2^** | **0.36** | | | | |

**Supplemental Table 6. Factorial design matrix and experimental results obtained for the control DoE (without silicone-CaO_2_)**

| Run | Factors | | Responses* | |
| --- | --- | --- | --- | --- |
|  | Islet seeding density (IEQ/device) | HEMOXCell concentration (µg/mL) | ATP content  (× 10^6^ RLU) | ATP/ LDH ratio  (× 10^6^ RLU/AU) |
| 1 | 1274 | 115 | 2.59 ± 1.19 | 3.31 ± 2.9 |
| 2 | 1274 | 435 | 2.86 ± 1.36 | 3.08 ± 3.04 |
| 3 | 6026 | 115 | 3.51 ± 1.25 | 0.36 ± 0.27 |
| 4 | 6026 | 435 | 3.35 ± 1.27 | 0.322 ± 0.2 |
| 5 | 300 | 275 | 0.73 ± 0.36 | 3.95 ± 3.35 |
| 6 | 3625 | 50 | 3.54 ± 1.06 | 0.69 ± 0.4 |
| 7 | 3625 | 500 | 3.72 ± 1.16 | 0.56 ± 0.36 |
| 8 | 7000 | 275 | 3.88 ± 1.57 | 0.37 ± 0.25 |
| 9 (central point) | 3625 | 275 | 3.09 ± 1.25 | 0.95 ± 0.6 |

***** Mean of seven independent experiments

**Supplemental Table 7. Estimated effects of factors and interactions and their statistical significance in the control optimization DoE (without silicone-CaO_2_)**

| Factors | ATP content  (× 10^6^ RLU) | | ATP/ LDH ratio  (× 10^6^ RLU/AU) | |
| --- | --- | --- | --- | --- |
|  | Estimated effect ± SD | p-value* | Estimated effect ± SD | p-value* |
| HEMOXCell concentration (HEM) | 0.12 ± 0.32 | 0.7079 | -0.16 ± 0.66 | 0.8084 |
| Islets’ seeding density (Islet) | **2.06 ± 0.33** | **< 10^-4^** | **-3.79 ± 0.66** | **< 10^-4^** |
| HEM & HEM | 1.18 ± 0.77 | 0.1310 | -0.26 ± 1.55 | 0.8643 |
| Islet &Islet | -1.47 ± 0.77 | 0.0631 | -2.82 ± 1,55 | 0.8852 |
| HEM & Islet | -0.430493 ± 0.65 | 0.5148 | 0.191397 ± 1.31 | 0.0757 |
| Blocks | **0.90 ± 0.49** | **< 10^-4^** | **-1,72 ± 0.99** | **0,0262** |

*p-values are computed from the analysis of variance performed for each response (Suppl. Tables 7 and 9) and statistical significance at 5% are highlighted in **bold**.

**Supplemental Table 8. Analysis of variance for ATP content from the optimization DoE without the silicone-CaO_2_ disk**.

| **Factors** | **Sum of squares**  **(× 10^11^)** | **Degrees of freedom** | **Mean square**  **(× 10^11^)** | **F ratio** | **p-value** |  |
| --- | --- | --- | --- | --- | --- | --- |
| HEMOXCell concentration (HEM) | 0.93 | 1 | 0.93 | 0.14 | 0.7079 |  |
| **Islet seeding density (Islet)** | **256.86** | **1** | **256.86** | **39,17** | **< 10^-4^** |  |
| HEM & HEM | 15.54 | 1 | 15.54 | 2.37 | 0.1310 |  |
| HEM & Islet | 23.86 | 1 | 23.86 | 3.64 | 0.0631 |  |
| Islet & Islet | 2.82 | 1 | 2.82 | 0.43 | 0.5148 |  |
| **Block** | **447.79** | **5** | **89.55** | **13.66** | **< 10^-4^** |  |
| Total error | 281.94 | 43 | 6.55 |  |  |  |
| **Determination coefficient R^2^** | **0.74** | | | | | |

**Supplemental Table 9. Analysis of variance for ATP/LDH ratio from the optimization control DoE without the silicone-CaO_2_ disk**.

| **Factors** | **Sum of squares**  **(× 10^11^)** | **Degrees of freedom** | **Mean square**  **(× 10^11^)** | **F ratio** | **p-value** |
| --- | --- | --- | --- | --- | --- |
| HEMOXCell concentration (HEM) | 1.57 | 1 | 1.57 | 0.06 | 0.8084 |
| **Islet seeding density (Islet)** | **867.16** | **1** | **867.161** | **32.71** | **< 10^-4^** |
| HEM &HEM | 0.78 | 1 | 0.78 | 0.03 | 0.8643 |
| HEM & Islet | 87.82 | 1 | 87.82 | 3.31 | 0.0757 |
| Islet & Islet | 0.55 | 1 | 0.55 | 0.02 | 0.8852 |
| **Block** | **377.48** | **5** | **75.49** | **2.85** | **0.0262** |
| Total error | 1.139 | 43 | 26.50 |  |  |
| **Determination coefficient R^2^** | **0.55** | | | | |

**Supplemental Table 10. Regression coefficients from optimization DoE**

| w/ silicone**-**CaO_2_ | | w/o silicone**-**CaO_2_ | | | | |  |
| --- | --- | --- | --- | --- | --- | --- | --- |
| Regression coefficients | ATP content  (RLU) | ATP/LDH ratio  (RLU/AU) | ATP content  (RLU) | |  | ATP/LDH ratio  (RLU/AU) |  |
| β_0_ | 3.02 × 10^6^ | 12.38 × 10^6^ | 1.62 × 10^6^ |  | | 4.71 × 10^6^ |  |
| β_A_ | -1.69 × 10^4^ | -2.56 × 10^4^ | -5.14 × 10^4^ |  | | 0.62 × 10^4^ |  |
| β_B_ | 2.19 × 10^4^ | 1.6 × 10^4^ | 0.86 × 10^4^ |  | | -1.52 × 10^4^ |  |
| β_AB_ | 0.77 | 4.23 | -0.28 |  | | -2.63 |  |
| β_AA_ | 29.54 | 11.45 | 11.74 |  | | 0.12 |  |
| β_BB_ | -0.14 | -0.46 | -0.06 |  | | 0.12 |  |
